# The Cost of the Sword: Escape Performance in Male Swordtails

**DOI:** 10.1371/journal.pone.0015837

**Published:** 2011-01-06

**Authors:** Alex Baumgartner, Seth Coleman, Brook Swanson

**Affiliations:** Biology Department, Gonzaga University, Spokane, Washington, United States of America; Ecole Normale Supérieure de Lyon, France

## Abstract

The handicap theory of sexual selection posits that male display traits that are favored in mate choice come at a significant cost to performance. We tested one facet of this hypothesis in the green swordtail (*Xiphophorus helleri*). In this species, the lower ray of male caudal fin is extended into a ‘sword’, which serves to attract potential mates. However, bearing a long sword may increase drag and thus compromise a male's ability to swim effectively. We tested escape performance in this species by eliciting C-start escape responses, an instinctive escape behavior, in males with various sword lengths. We then removed males' swords and retested escape performance. We found no relationship between escape performance and sword length and no effect of sword removal on escape performance. While having a large sword may attract a predator's attention, our results suggest that sword size does not compromise a male's escape performance.

## Introduction

Males in many species have elaborate display traits that they use to attract females [1]. The handicap theory of sexual selection [2], [3] states that while such traits may increase a male's attractiveness, they may also come with significant costs [4]. Exaggerated visual ornaments may make males more conspicuous to females, while also increasing their conspicuousness to eavesdropping predators [1], [5]–[7]. In guppies, females prefer brightly colored males as mates [8], but so do predatory cichlids [9]. Similarly, in the barn swallow *Hirundo rustica*, males with elongated tail feathers are preferred by females but are also less efficient at capturing prey [10]. In addition to increasing the likelihood of detection by predators or potential prey, exaggerated male display traits may also inhibit a male's ability to escape predators: the drag imposed by moving a large display trait through the medium—water or air—may significantly reduce escape velocity [11], [12].

In this study, we examined the relationship between escape response swimming performance and an elongated male display trait in the green swordtail, *Xiphophorus helleri*. The lower rays of male *X. helleri* caudal fins have been extended into an elongated ornament—the sword—which is assessed by females in mate choice. Females prefer males with the longest swords as mates [13]. This is a preexisting bias that stems from a general preference for large apparent size [14]. Females do not possess any ornamental fin decoration or extension. Natural variation exists both in body length and sword length [14], so it is expected that males with longer (and possibly more decorative) swords will have a higher chance of reproductive success, since they are more likely to be chosen by females. However, it is unclear how the sword affects male swimming performance and may be affected by natural selection.

Swordtails as a group are commonly used as a model for studying the conflict between sexually selected and naturally selected traits. However, data on the cost of the sword to males is complex and somewhat contradictory. For instance, Basolo and Alcaraz [15] found that oxygen consumption rates are higher in *X. montezumae* males with intact swords compared to males with experimentally shorted swords, during both steady swimming and courtship swimming. This suggests that while females prefer the sword, it comes at a metabolic cost to the male and shorter or nonexistent swords may be preferred by natural selection. On the other hand, Royle *et al*. [16] demonstrated that average speed during escape responses is higher in *X. helleri* males with naturally longer swords when the effects of size are removed. This result has two possible explanations. One is that the sword confers a swimming cost, but that males are able to compensate, suggesting that sword length is a good indicator of male quality. Alternatively, increased sword length may somehow actually improve fast start performance. Here, we wish to explore this relationship between the sword and the escape response further.

The C-start escape response is the primary maneuver used by teleost fishes to escape predators [17]. During a predator's attack, the prey fish responds to changes in pressure in the water, which cause an activation of the large Mauthner neurons [18]. These Mauthner neurons activate motor neurons along the side of the fish's body opposite the stimulus. The muscles here contract almost simultaneously, causing the fish to bend into a “C” shape. In the second part of the maneuver, the C-shaped bend moves towards the caudal fin and the fish propels itself away from its initial position [18], [19]. A faster C-start greatly improves a fish's chances of evading a predator [20], [21]. Thus we believe that C-start performance should be a good indicator of a swordtail's ability to escape predation. Furthermore, swordtail habitat is variable, but generally includes slow moving streams and pools. Therefore, we believe that unsteady swimming performance should be important in determining male swordtail survival (Coleman pers. obs.).

We tested the hypothesis that sword length variation predicts variation in escape response performance in male green swordtails, *X. helleri* (either positively or negatively). First we examine the effect of variation in sword length. If the sword increases drag, males with shorter swords should perform better than males with longer swords in one or several of the escape performance variables we measured. Alternatively, if there is no cost or fish are able to compensate for the increased drag, there should be no relationship or a positive relationship between sword length and escape performance. Additionally, we removed the swords from all of the fish. If the sword imposes a drag cost, removal should improve escape performance. Furthermore, if there is compensation, fish with longer swords should demonstrate more improvement than short sworded individuals.

## Materials and Methods

### Ethics Statement

All experimental procedures were carried out in accordance with the recommendations of the Gonzaga University Institutional Animal Care and Use Committee (IACUC), who approved the study (authorization number A-1009071).

### Experimental Set-up

**Figure 1:**
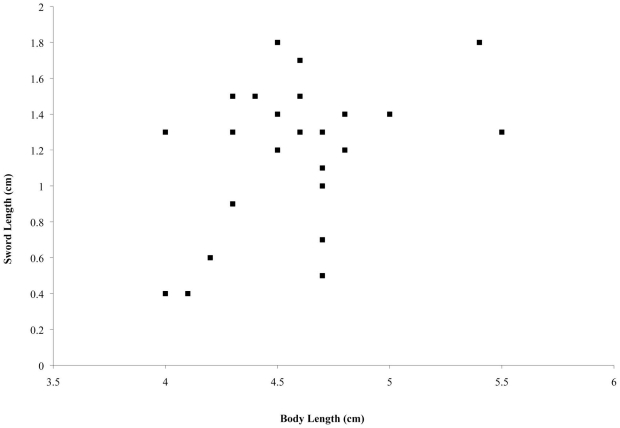
Variation in both body length and sword length. Sword length, measured from the extension of the sword from the caudal fin to the tip of the sword, is shown against body length, measured from the tip of the mouth to end of the caudal fin (excluding the sword). We used fish of both long and short body and sword lengths. Body length: mean  = 4.58 cm, std. err.  = 0.08 cm, range  = 5.1 cm. Sword length: mean  = 1.18 cm, std. err.  = 0.08 cm, range  = 1.4 cm. There is a slight positive relationship between fish length and sword length (R^2^ = 0.16).

Twenty-four fish of the species *Xiphophorus helleri* were obtained from commercial vendors. Only healthy, mature males were selected, but considerable variation in sword length and overall body size still existed (Figure 1, Table 1).

**Table 1:** Morphological and performance data for the 24 *X. helleri* males used in this study.

			Absolute Max. Velocity (m/s)		Propulsive Tail Stroke Velocity (m/s)		Distance Traveled (m)		Time to Max. Velocity (s)	
Fish	Body Length (cm)	Sword Length (cm)	Average	Std. Dev.	Average	Std. Dev.	Average	Std. Dev.	Average	Std. Dev.
1	4.0	0.4	1.120	0.128	0.610	0.204	0.036	0.003	0.015	0.002
2	4.3	1.3	1.324	0.044	0.658	0.175	0.043	0.009	0.018	0.002
3	4.3	0.9	1.180	0.075	0.760	0.250	0.037	0.005	0.018	0.003
4	4.5	1.2	1.168	0.069	0.794	0.378	0.034	0.002	0.016	0.002
5	4.7	1.1	1.068	0.083	0.706	0.072	0.045	0.002	0.019	0.001
6	5.0	1.4	1.400	0.150	0.600	0.146	0.037	0.005	0.015	0.002
7	4.6	1.3	1.167	0.081	0.615	0.195	0.034	0.008	0.017	0.003
8	4.4	1.5	1.160	0.097	0.754	0.086	0.033	0.006	0.018	0.001
9	4.8	1.4	1.458	0.047	0.618	0.219	0.037	0.008	0.015	0.001
10	5.5	1.3	1.306	0.050	0.562	0.057	0.047	0.011	0.018	0.003
11	4.8	1.2	1.208	0.045	0.574	0.073	0.040	0.004	0.017	0.002
12	4.7	1.3	1.228	0.027	0.831	0.099	0.038	0.006	0.018	0.004
13	4.1	0.4	0.885	0.144	0.315	0.007	0.022	0.004	0.013	0.003
14	4.6	1.7	0.975	0.169	0.485	0.049	0.030	0.008	0.013	0.002
15	4.5	1.8	0.831	0.154	0.683	0.308	0.042	0.008	0.014	0.006
16	4.2	0.6	0.508	0.234	0.320	0.127	0.030	0.004	0.010	0.003
17	4.7	0.5	1.025	0.038	0.667	0.313	0.028	0.005	0.011	0.003
18	5.4	1.8	1.232	0.155	0.487	0.055	0.042	0.014	0.016	0.007
19	4.5	1.4	0.818	0.111	0.520	0.071	0.034	0.009	0.014	0.001
20	4.3	1.5	1.068	0.068	0.533	0.186	0.035	0.007	0.014	0.005
21	4.7	0.7	1.173	0.025	0.843	0.110	0.042	0.004	0.015	0.002
22	4.0	1.3	0.820	0.106	0.667	0.160	0.033	0.008	0.013	0.002
23	4.6	1.5	1.180	0.127	0.560	0.131	0.039	0.009	0.014	0.004
24	4.7	1.0	0.545	0.078	0.250	0.014	0.021	0.003	0.010	0.002

Performance data were recorded with intact swords. Individual values are averages of the three fastest C-starts (of approximately 10 recorded per fish).

Fish were housed in 10 or 20-gallon aquaria. To prevent overcrowding, two fish were kept in each 10-gallon tank and no more than three fish were kept in each 20-gallon tank. Partial water changes were performed weekly to maintain the quality of the water. Fish were fed Tetramin® Tropical Fish Flakes once per day *ad libitum*. Fish were also fed frozen brine shrimp approximately once per week. Each individual was photographed and documented so that the length of its sword could be measured. Each fish was allowed to acclimate for between 7 and 10 days before any testing was conducted, to minimize stress.

### Performance Testing and Sword Removal

Each fish's C-start performance was tested before its tail was removed. Fish were tested multiple times over a five-day period, to ensure that a maximum effort for each fish was observed. Each fish was tested on three different days and was allowed to rest for at least 24 hours before being tested again.

To obtain a C-start escape, the fish was placed in a small plastic tub containing water 3–4 cm deep, with 1-cm grid paper set at the bottom of the tub for reference. The water was kept at room temperature (approximately 22°C, the same temperature in which the fish were housed) during the testing. Fewer than 10 C-start videos were obtained at one time, so that the lamp used to illuminate the tub would not heat the water, and the lamp was turned off between tests. A Phantom® V-5.1 high-speed camera (Vision Research, Wayne, NJ, USA), recording at 600 frames per second, was used to document the fish's swimming. The fish was allowed to swim to the middle of the tub, so that it would not collide with the tub's edge during the C-start escape. A small glass rod was then thrust rapidly down into the water in front of the fish. This stimulus is similar to a predator's attack and causes the fish to reflexively swim away from the perceived threat [17]. This was repeated until two or three videos of high-quality C-starts were obtained. Thus, over the five-day testing period, approximately 9 videos of C-start escapes were recorded for each fish.

**Figure 2:**
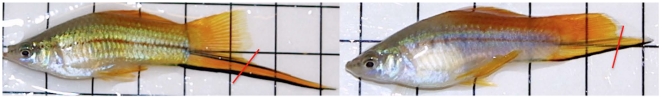
Variation in sword length. Two *X. helleri* males used in our study; one displaying an exceptionally long sword (left) and the other a very short sword (right), despite similar body lengths. Swords were removed at the point where the sword extends beyond the caudal fin, as indicated by the red lines.

Following the initial round of performance testing, the swords of each fish were removed and the testing procedure was repeated, allowing us to compare each individual's performance without its sword to its baseline performance. Swords were removed with a razorblade, at the point where the sword extends beyond the rest of the caudal fin (Fig. 2). After removal of the sword, fish were allowed one week to recuperate before testing began again. Following the recovery period, fish were tested once more over another five-day period using the same procedure as in the initial round of testing. Sham surgeries were not conducted for several reasons. Firstly, all of the fish in the experiment were captured and photographed prior to initial testing, procedures that closely mimicked the sword removal treatment, save the sword removal. Therefore, the initial testing is more like a sham treatment then a set of completely untreated fish. Secondly, in previous experiments there was no effect of sham sword removal on swordtail swimming (removal of 1 mm of sword) [15]. Finally, our results suggested that sword removal has no effect on swimming: see below.

### Data Analysis

For each round of performance testing (before sword removal and after), the three videos that best represented the individual's maximum effort (based on speed and distance traveled) were chosen for each fish. These videos were digitized and analyzed separately using Phantom® 640 software. For each video, a single point on the dorsal side of the fish between its eyes was tracked throughout the C-start behavior. The start of the behavior was chosen as when the fish first began to bend into a “C” shape, and the end was defined as the instant when the tail, during its first propulsive stroke, passed through the midline of the body [16].

Escape performance variables examined were: maximum instantaneous velocity (usually during stage 1 of the C-start), maximum instantaneous velocity during the propulsive tail stroke (stage 2), average velocity throughout the behavior, distance traveled during the behavior, time taken to complete the behavior, and time taken to reach the absolute maximum velocity (a measure of average acceleration). Videos were analyzed separately and performance variables were averaged to produce one data point for each performance variable per fish. We found variability in maximum performances within individuals, however performances were repeatable and inter-individual variation was larger than intra-individual variation (Table 1).

First, performance data was compared against sword length using regression analysis, for all of the escape variables measured. Sword length is weakly correlated with fish total length (p = 0.05). Therefore the regression analysis was also repeated using body lengths per second for speed variables. However, this did not change the conclusions (data not shown) and un-scaled velocities were used in the rest of the analysis. Secondly, performance variables recorded after sword removal were regressed against original sword length. Third, multivariate ANOVA (MANOVA) was used to assess performance differences for all fish before and after removal of the sword. Finally, differences between individual escape performance variables were assessed *post hoc* using Tukey HSD tests. All statistical analyses were conducted in JMP 8 (SAS Institute Inc. Cary, NC, USA).

## Results

**Figure 3:**
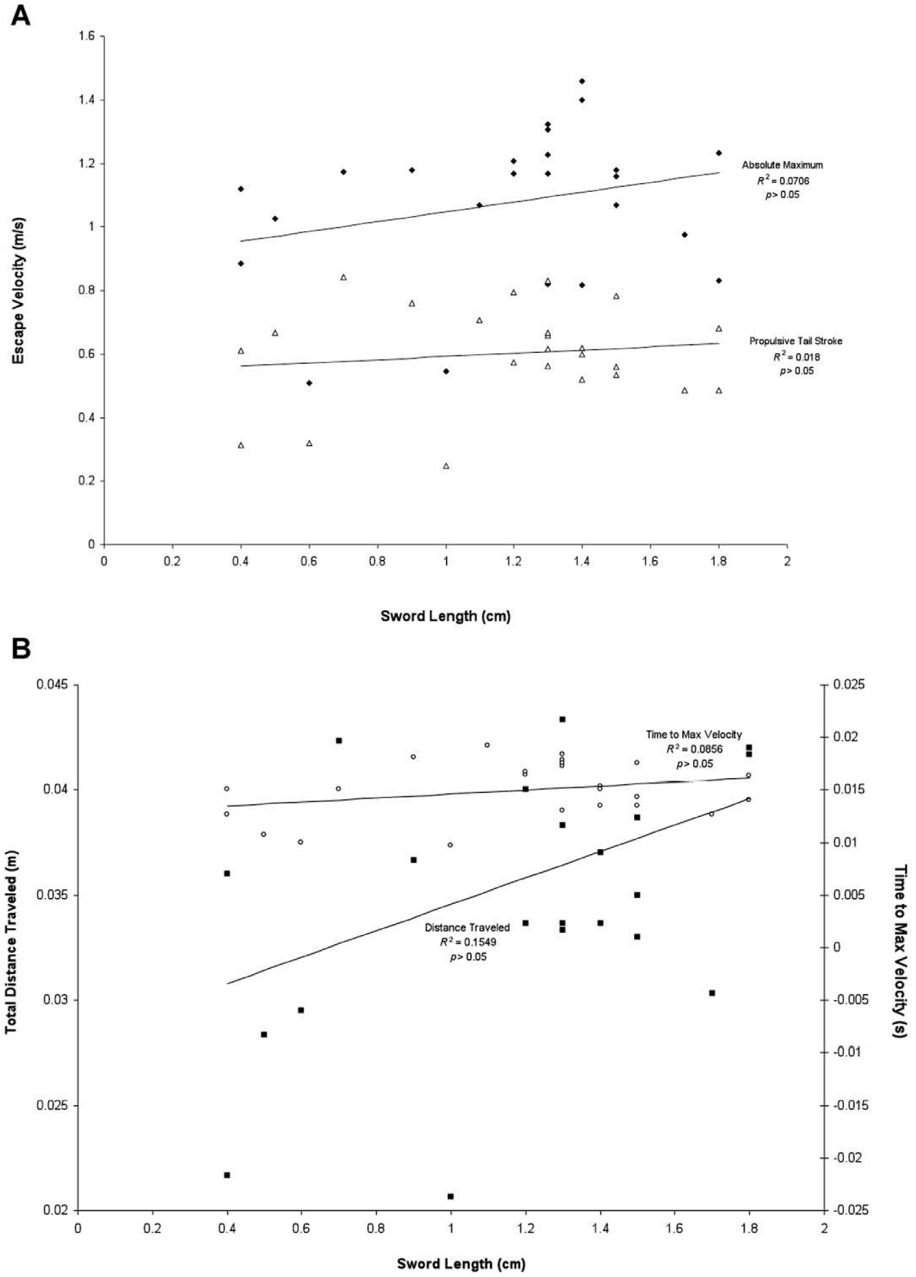
The effect of sword length on C-start escape performance. In panel A, two measures of escape velocity are shown for each individual against the length of its sword: absolute maximum velocity (indicated by the solid diamonds) and maximum velocity during the propulsive tail stroke (indicated by the hollow triangles). In panel B, two other measures of performance are shown against the length of the sword: the time taken to reach the fish's maximum velocity (indicated by hollow circles) and total distance traveled during the behavior (indicated by solid squares). Based upon extremely low *R* ^2^ values and non-significant *p*-values, it can be inferred that changes in escape performance are not associated with varying sword lengths. Thus it does not appear that sword length has any significant effect on escape performance.

**Figure 4:**
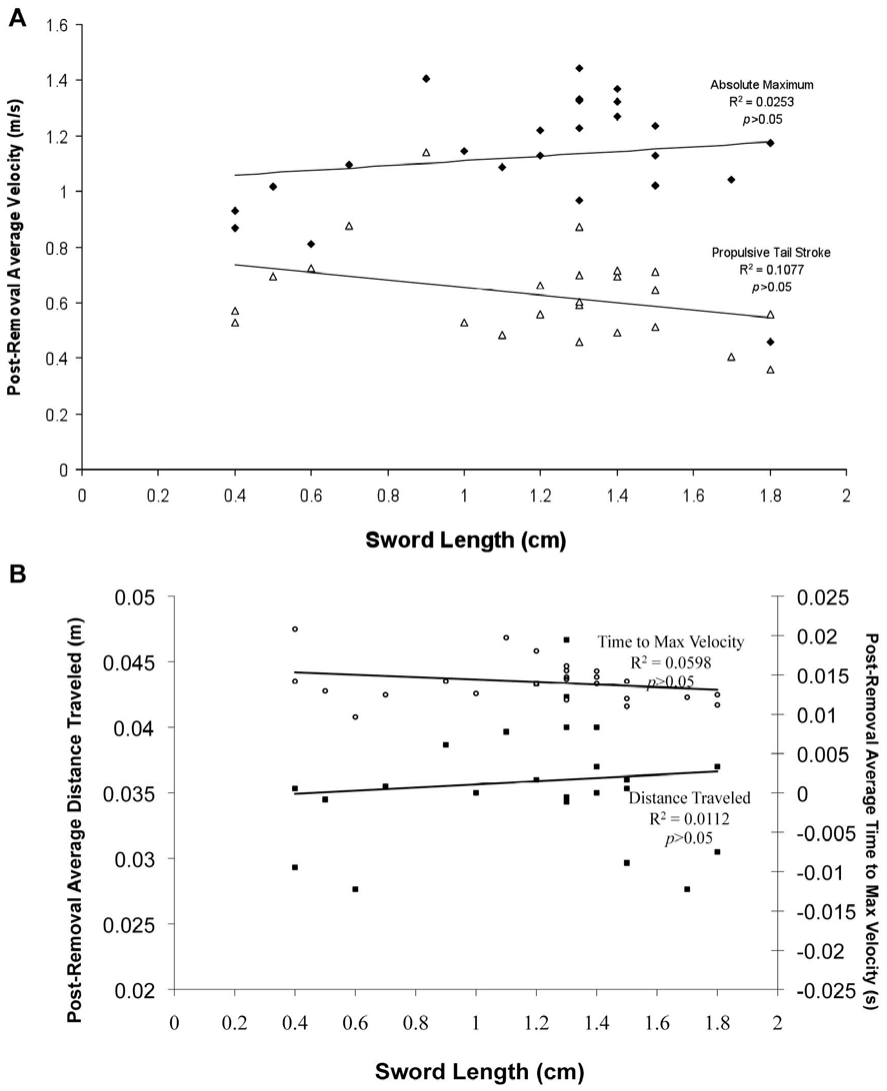
Males with long swords are not inherently better performers. As in Figure 2, four escape performance variables are shown for each individual against the length of its sword. However, here we compare the fish's escape performance after its sword was removed to its original sword length. Panel A shows two post-removal measures of escape velocity—absolute maximum velocity (stage 1) and maximum velocity during the propulsive tail stroke (stage 2)—against the original length of the individual's sword. Panel B shows two other post-removal measures of escape performance—total distance traveled and time taken to reach the maximum velocity—against the original length of the sword. Low *R* ^2^ values indicate that individuals with longer swords are not inherently better at performing escape response maneuvers than those with short swords.

We found no significant relationship between sword presence or length and escape performance. Sword length was not a predictor of escape velocity (Figure 3) or any of the other escape performance variables we measured. For instance, the total distance traveled by a fish during the escape maneuver and the time taken to reach the fish's maximum velocity were not associated with the length of its sword (Figure 3). When the analysis was repeated using length specific velocities, the conclusions were the same (data not shown). In the regressions of sword-free performance against sword length, there were still no effects associated with sword length (Figure 4). Even after their swords were removed, individuals who originally had longer swords did not perform significantly differently than individuals whom originally possessed shorter swords. This indicates that fish with longer swords are not intrinsically better escape performers than fish with short swords.

**Figure 5:**
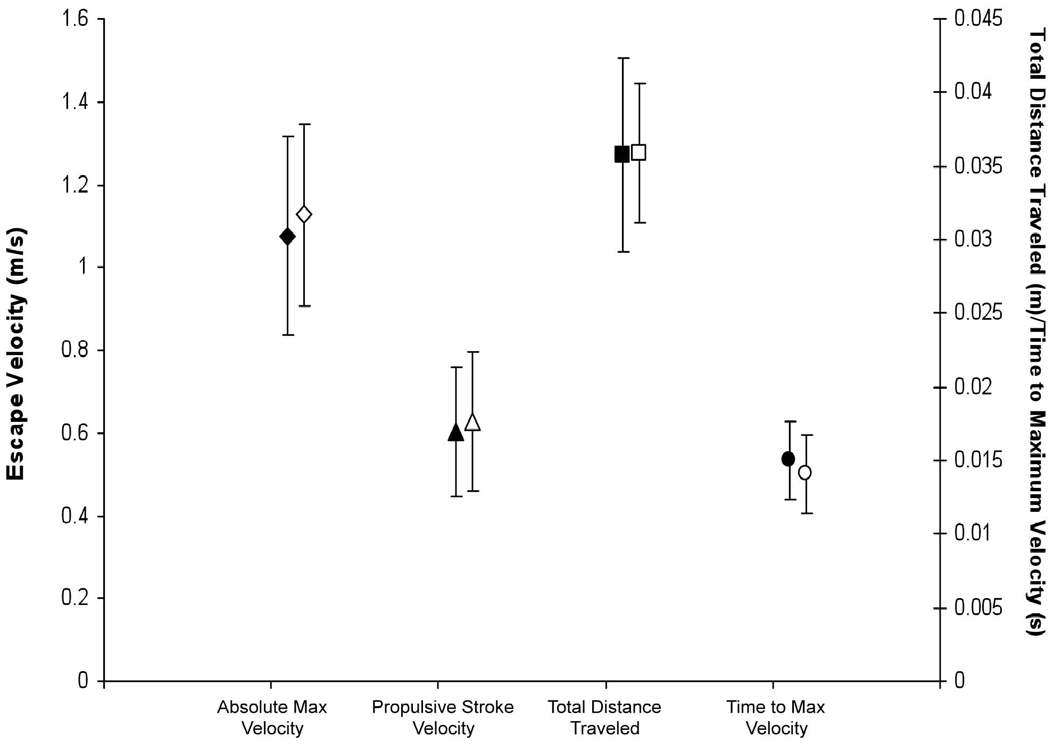
A comparison of performance before and after removal of the sword. Overall C-start escape performance is shown as an average for all individuals. Solid markers represent averages before the swords were removed, while hollow markers represent averages after removal. The same four performance variables are compared as in Figs. 3 and 4. whiskers represent ±1 standard deviation. There is no significant change in any performance variable after the sword is removed.

Removal of the sword also was not associated with any change in performance (Figure 5). The MANOVA and post hoc tests all had p-values greater than 0.05. This indicates that the variation within the pre removal and post removal groups was much larger than the difference between groups.

## Discussion

Well-established theories of sexual selection maintain that traits which are favorable in mate choice will be strongly selected for, even if natural selection does not favor these traits. Zahavi and Zahavi [3] argued that such traits might actually bear a significant cost to the individual. In this case the ability to bear an exaggerated trait may be an accurate measure of quality [16]. Males also may be able to compensate for the cost associated with theses exaggerated traits and therefore not suffer decreased performance [11].

Our results suggest that in *Xiphophorus helleri* the sword does not compromise male escape performance. We found that escape performance is variable, but is not correlated with sword length. This performance variation may be driven by variation in body shape or muscle physiology, but seems to be decoupled from sword size. It is well known that *X. helleri* females prefer males with longer swords [13], but the cost of the sword in relation to escape performance seems to be negligible. There are two possible explanations for this pattern. First, the hydrodynamics of the escape response may be insensitive to the presence of a sword. Recent work has suggested that body and caudal fin depth (rather than length variables) are good predictors of escape performance [16], [22]. In addition, the frictional drag of the sword during such a rapid and unsteady behavior as the C-start could be negligible. This possibility suggests swords may be evolutionarily ‘ideal’ ornaments. If males with swords gain attractiveness with little increase in predation risk, then swords would be free to evolve into the highly exaggerated ornaments typical of the genus [23], [24]. However, this hypothesis would have to be tested by looking at populational and species differences in sword length and performance. The second possible explanation is that males with long swords are able to compensate physiologically, so that although they have higher drag associated with the escape response, they show no decrease in performance [11], [16]. If this were the case we would expect those fish with longer swords to have increased improvement upon removal of the sword. However, we found no improvement in performance upon removal of the sword in any fish, and pre-removal sword length was still not correlated with escape performance after removal.

Our results differ slightly from Royle *et al.* [16], who found a slight positive correlation between sword length/body depth and escape response performance. This could be due to differences in methods or perhaps sample size. However, the key conclusion—that the sword does not seem to hamper escape performance—is the same. Royle *et al.* [16] suggest that their data could be explained by long-sworded males being of higher quality, and therefore being able to compensate for the extra sword length. Our data (both correlative and experimental) suggests that the sword is a neutral trait in terms of escape response performance.

Still, we do not believe that the sword is cost-free altogether. Basolo and Alcaraz [15] demonstrated that the sword increases the energetic cost of both steady and courtship swimming. The sword also certainly bears some metabolic cost during development. The size and coloration of the sword are particularly attractive to predators [14], so that they may have to endure more attacks, but they should be equally good at surviving these attacks [20]. Because the natural habitat of these fish is relatively still water (Coleman pers. obs.), we believe that escape response performance is a better measure of swimming cost than steady swimming. Although there may be a significant metabolic cost to having a large sword in steady swimming, if most swordtail swimming is unsteady, the swimming cost associated with the sword may be slight.

Based on our findings, a complex picture of the costs associated with bearing a sword emerges. We know that the sword is advantageous in sexual selection [13], [14] and that it also increases the costs associated with steady swimming [15]. Furthermore, there must be at least some metabolic cost to growing a long sword [16]. However, we found no relationship between sword presence or size and escape performance. Our data, the data of Royle *et al.* [16] and that of Langerhans [22] suggest that large caudal regions or extensions, such as the sword, do not inflict a severe handicap on the fish. If the impetus for the evolution of this sexually selected ornament were a preexisting female preference for larger size [13], then it would be possible for a variety of ornaments to elicit and exploit this female preference. However, there should be intense natural selection removing males from the population that have ornaments that compromise their ability to escape from predators. Here we see multiple evolutions of a specific, large, conspicuous ornament [25], [26] that seems to have little effect on the escape swimming ability of the bearer. This is in fact is exactly what we would expect selection to produce—an ornament that exploits a female preference, yet should not increase predation.

## References

[1] Andersson M (1994). Sexual Selection..

[2] Zahavi A (1975). Mate selection—a selection for a handicap.. J Theor Biol.

[3] Zahavi A, Zahavi A (1997). The handicap principle: A missing piece of Darwin's puzzle..

[4] Garcia CM, Jimenez G, Contreras B (1994). Correlational evidence of a sexually-selected handicap.. Behav Ecol Sociobiol.

[5] Endler JA (1992). Signals, signal conditions, and the direction of evolution.. Am Nat.

[6] Zuk M, Kolluru GR (1998). Exploitation of sexual signals by predators and parasitoids.. Q Rev Biol.

[7] Kotiaho JS (2001). Costs of sexual traits: A mismatch between theoretical considerations and empirical evidence.. Biol Rev.

[8] Houde AE (1997). *Sex, color, and mate choice in guppies*..

[9] Godin JGJ, McDonough HE (2003). Predator preference for brightly colored males in the guppy: a viability cost for a sexually selected trait.. Behav Ecol.

[10] Moller AP, de Lope F (1994). Differential costs of a secondary sexual character: an experimental test of the handicap principle.. Evol.

[11] Oufiero CE, Garland T (2007). Evaluating performance costs of sexually selected traits.. Funct Ecol.

[12] Weihs D (1989). Design features and mechanics of axial locomotion in fish.. Am Zool.

[13] Rosenthal GG, Evans CS (1998). Female preference for swords in *Xiphophorus helleri* reflects a bias for large apparent size.. Proc Natl Acad Sci.

[14] Rosenthal GG, Flores Martinez TY, Garcia de Leon FJ, Ryan MJ (2001). Shared preferences by predators and females for male ornaments in swordtails.. Am Nat.

[15] Basolo AL, Alcaraz G (2003). The turn of the sword: length increases male swimming costs in swordtails.. Proc Biol Sci.

[16] Royle NJ, Metcalfe NB, Lindstrom J (2006). Sexual selection, growth compensation and fast-start swimming performance in Green Swordtails, *Xiphophorus helleri*.. Funct Ecol.

[17] Domenici P, Blake RW (1997). The kinematics and performance of fish fast-start swimming.. J Exp Biol.

[18] Nissanov J, Eaton RC (1989). Reticulospinal control of rapid escape turning maneuvers in fishes.. Am Zool.

[19] Wakeling JM (2006). Fast-start mechanics.. Fish biomechanics: Fish physiology.

[20] Walker JA, Ghalambor CK, Griset OL, McKenney D, Reznick DN (2005). Do faster starts increase the probability of evading predators?. Funct Ecol.

[21] Katzir G, Camhi JM (1993). Escape response of black mollies (*Poecilia sphenops*) to predatory dives of a pied kingfisher (*Ceryle rudis*).. Copeia.

[22] Langerhans RB (2009). Morphology, performance, fitness: Functional insight into a post-Pleistocene radiation of mosquitofish.. Biol Lett.

[23] Rauchenberger M, Kallman KD, Morizot DC (1990). Monophyly and geography of the Panuco Basin swordtails (genus *Xiphophorus*) with descriptions of four new species.. Am Mus Nat Hist Novitates.

[24] Basolo AL (1995). A further examination of a pre-existing bias favoring a sword in the genus *Xiphophorus*.. Anim Behav.

[25] Morris MR, de Queiroz K, Morizot, DC (2001). Phylogenetic relationships among populations of northern swordtails (*Xiphophorus*) as inferred from allozyme data.. Copeia.

[26] Marcus JM, McCune AR (1999). Ontogeny and phylogeny in the northern swordtail clade of *Xiphophorus*.. Syst Biol.

